# Current MUAC Cut-Offs to Screen for Acute Malnutrition Need to Be Adapted to Gender and Age: The Example of Cambodia

**DOI:** 10.1371/journal.pone.0146442

**Published:** 2016-02-03

**Authors:** Marion Fiorentino, Prak Sophonneary, Arnaud Laillou, Sophie Whitney, Richard de Groot, Marlène Perignon, Khov Kuong, Jacques Berger, Frank T. Wieringa

**Affiliations:** 1 Institut de Recherche pour le Developpement, UMR 204, IRD/Montpellier1/Montpellier2/SupAgro (Nutripass), Montpellier, France; 2 National Nutrition Program, Maternal and Child Health Center, Phnom Penh, Cambodia; 3 UNICEF, Maternal Child Health and Nutrition section, Phnom Penh, Cambodia; 4 World Food Program, Phnom Penh, Cambodia; 5 Independent consultant, Phnom Penh, Cambodia; 6 Ministry of Agriculture, Forestry and Fisheries, FiA Administration DFPTQ, Phnom Penh, Cambodia; Vanderbilt University, UNITED STATES

## Abstract

**Background:**

Early identification of children <5 yrs with acute malnutrition is a priority. Acute malnutrition is defined by the World Health Organization as a mid-upper-arm circumference (MUAC) <12.5 cm or a weight-for-height Z-score (WHZ) <-2. MUAC is a simple and low-cost indicator to screen for acute malnutrition in communities, but MUAC cut-offs currently recommended by WHO do not identify the majority of children with weight-for-height Z-score (<-2 (moderate malnourished) or r<-3 (severe malnourished). Also, no cut-offs for MUAC are established for children >5 yrs. Therefore, this study aimed at defining gender and age-specific cut-offs to improve sensitivity of MUAC as an indicator of acute malnutrition.

**Methods:**

To establish new age and gender-specific MUAC cut-offs, pooled data was obtained for 14,173 children from 5 surveys in Cambodia (2011–2013). Sensitivity, false positive rates, and areas under receiver-operator characteristic curves (AUC) were calculated using wasting for children <5yrs and thinness for children ≥5yrs as gold standards. Among the highest values of AUC, the cut-off with the highest sensitivity and a false positive rate ≤33% was selected as the optimal cut-off.

**Results:**

Optimal cut-off values increased with age. Boys had higher cut-offs than girls, except in the 8–10.9 yrs age range. In children <2yrs, the cut-off was lower for stunted children compared to non stunted children. Sensitivity of MUAC to identify WHZ<-2 and <-3 z-scores increased from 24.3% and 8.1% to >80% with the new cut-offs in comparison with the current WHO cut-offs.

**Conclusion:**

Gender and age specific MUAC cut-offs drastically increased sensitivity to identify children with WHZ-score <-2 z-scores. International reference of MUAC cut-offs by age group and gender should be established to screen for acute malnutrition at the community level.

## Introduction

Wasting or acute malnutrition is a major contributor to the global disease burden and to child mortality. In 2011, >50 million or 8% of all children <5y (years) were affected [1, 2]. Acute malnutrition can be divided into severe acute malnutrition, being defined as a weight-for-height Z-score (WHZ) <-3 or a mid-upper arm circumference of <11.5 cm, or moderate acute malnutrition, with a WHZ between -2 and -3 Z-scores or a MUAC (mid-upper-arm circumference) between 11.5 and 12.5 cm. Severe acute malnutrition affected 19 million children in 2011, causing an estimated 500,000 deaths, which represents ~7.5% of all<5y mortality [1].

For early identification of children with acute malnutrition, an easy, accurate, and low-cost indicator is needed. The golden standard to identify acute malnutrition is weight-for-height z-scores [3]. Unfortunately, scales and/or height board are not always available for screening at community level in many developing countries. Therefore, as subcutaneous fat and muscle mass decrease in undernourished children, MUAC has been used as a proxy indicator to screen for acute malnutrition. Moreover, MUAC was shown to predict child mortality at least as well as WHZ [4–6]. In the revised (2013) guidelines for the management of severe acute malnutrition, the World Health Organization (WHO) recommends using 11.5cm and 12.5cm as cut-offs for admission and discharge criteria for severe and moderate acute malnutrition in children under 5y respectively [7]. However, recently we showed that MUAC and WHZ identify different groups of children with malnutrition, and that a cut-off of 13.3 cm, rather than 11.5 cm would increase sensitivity of MUAC to identify children with a WHZ<-2 or <-3 [8].

However, it is questionable whether using only 1 cut-off for all children between 6 and 59 months of age is valid, as MUAC was shown to be age and gender dependent in several studies [9, 10]. Recent research has highlighted the problems with using one, unique cut-off for MUAC for identifying malnutrition, as it respectively over- and underestimates malnutrition in children under and above 2 years old [10, 11], and identifies more girls than boys with malnutrition. Onis et al (1997) developed a MUAC-for-age z-scores reference based on US data [11]. However, this reference requires an accuracy of the child's age of 1 month. At community level, it is often very difficult to estimate age precisely and a table reference is then needed to verify whether the child is malnourished or not. The latest WHO guidelines for the treatment of severe acute malnutrition recommend refining the cut-offs for MUAC to identify acute malnutrition for different groups in children under 5 y, using perhaps also different cut-offs for stunted and non-stunted children, and establishing cut-offs for children <6 months or >5 y [7]. Malnutrition in the latter age group is important to address also, even though it is less linked to mortality. Acute malnutrition during school years can impair physical and mental development [2], and acute malnutrition is widely spread also in school-aged children. For example, one third of Southeast Asian school children are affected by thinness [12].

The present study aimed to answer several research questions posed in the revised WHO guidelines for the treatment of SAM, especially focusing on the discrepancy between MUAC and WHZ, defining new MUAC cut-offs adapted to age and gender in children under 5 y, and MUAC cut-offs for older children. We used pooled data from several surveys carried out in Cambodia between 2011 and 2013. Cambodia is one of the 42 countries with the highest child mortality rates [13], and prevalence of acute malnutrition is high with 10% of <5y children having a weight-for-height Z-score (WHZ) <-2 [14].

The main objectives of the study was to find new cut-off values for MUAC in children from 0 to 14 y based on age groups of 0.5–2 y, 2–5 y, 5–8 y, 8–11 y and 11–14 y, to identify WHZ-scores of <-2 and -3 Z-scores respectively, and to verify whether there were gender differences or differences between stunted and non-stunted children respectively.

## Methods

Data on weight, height, MUAC, gender and age were available for a total of 14,157 Cambodian children, from 5 different surveys conducted in Cambodia by several organizations (UNICEF; WHO; WFP; World Vision; International Relief and Development; Institut de Recherche pour le Développement) between 2011 and 2013. All parents of participants gave their written consent. All studies were approved by the National Ethic Committee for Health Research (NECHR) of the Ministry of Health, Phnom Penh, Cambodia. In 4 studies, the target population was children aged 0–4.9 y whereas in 1 study children aged 5–14 years were included.

Measurements of MUAC (to the nearest 1 mm) were made using a non-stretch tape measure (provided through UNICEF) in all studies. Weight was measured with the child wearing only light clothes, and was recorded to the nearest 0.1 kg by a Salter scale in the 4 studies on children under 5y and by a Seca scale in the study on children above 6 y. Length was measured in children under 2 yrs and height was measured in children above 2 yrs. Length/height was measured to the nearest 0.1 cm with a Holtain infantometer in children under 6 y and with a locally produced stadiometer in children above 6 y. Height boards and scales were daily calibrated. Anthropometric teams were standardized [15]. Age was determined (calculated in the nearest months) by asking of both the child’s age and date of birth to mothers of children under 6 y. In the study on children above 6 y, date of birth was collected from school registers. Data were entered into PASW statistics 18 (Chicago, USA) or Epidata Entry (EpiData Association, http://www.epidata.dk). Weight for Height z-scores (WHZ), Height for Age z-scores (HAZ), Body Mass Index for Age z-scores (BAZ) were calculated from anthropometric data using WHO AnthroPlus v1.0.4. As recommended by WHO, and using the WHO child growth references [16], acute malnutrition was defined in children under 5 y by respectively WHZ<-3 z-score (severe acute malnutrition) and WHZ between -3 and -2 z-scores (moderate acute malnutrition), and in children above 5 y by respectively BAZ<-3 z-scores (severe acute malnutrition) and BAZ between -3 and -2 z-scores (moderate acute malnutrition). Stunting was defined as a HAZ<-2 z-scores.

To assess the performance of MUAC cut-offs compared to the golden standard recommended by WHO to define severe and moderate acute malnutrition), receiver operating characteristic curves (ROC curves) were constructed. The sensitivity and false positive rates (1-specificity) of MUAC were determined using wasting (WHZ<-2 z-score in children under 5 y) and thinness (BAZ<-2 z-score in children above 5 y) as gold standards of acute malnutrition. The ROC curve is the plot of sensitivity versus false positive rate of MUAC cut-offs. The area under curve (AUC) is the area between the curve and the segment (0,0) and (1,1), which corresponds to a random classifier. A larger AUC indicates a more accurate diagnosis of acute malnutrition defined by WHZ cut-offs [17]. Data analysis was performed using SPSS version 20.0 (SPSS, Inc., Chicago, IL).

ROC curves were constructed for a large set of MUAC cut-offs, increased by steps of 1 mm, in order to find the new cut-off for severe and moderate acute malnutrition respectively. We generated a dummy variable for each MUAC cut-off, and did this for each MUAC-value in steps of 1 mm. Then, we calculated the AUC for that dummy variable in comparison to the golden standard, using the same methodology as Laillou et al and Fernandez et al [8, 18]. In order to evaluate the performance of our analysis, the corresponding Youden index, which is the difference between the true positive rate (sensitivity) and the false positive rate, was calculated: 1 indicating a perfect test, and 0 a useless test [19]. These analyses were conducted overall, and by gender and age groups. Several criteria were chosen to select new cut-off presented in this paper. The best correspondence between 2 indicators was first defined as the MUAC cut-off with highest AUC in the ROC curve. However, the sensitivity which one wants to be optimized for screening purposes, in order to miss a minimum of children with acute malnutrition, increased considerably around the MUAC cut-off with the highest AUC, whereas the AUC hardly changed (cf. S1–S5 Figs). Thus, among the highest values of AUC (within 0.02 from highest AUC), the cut-off with the highest sensitivity was selected. As this can lead to many false-positive children to be included after screening, a third criteria was introduced by the authors to define the new cut-off: a false positive rate below 1/3 of non-malnourished children. To summarize, among the highest values of the area under the curve (highest AUC -0.02), the cut-off with the highest sensitivity and a false positive rate of less than 33% was considered to be the new optimal cut-off. In addition, we calculated the difference of Youden index between the new optimal cut-off and the cut-off obtaining the highest Youden index. These calculations were done for 0.5–2 y, 2–5 y, 5–8 y, 8–11 y and 11–14 y, for girls, boys and all. Calculations were repeated for stunted and non stunted children under 5 y respectively [7].

## Results

In total, data was available for 14,157 children (51% male), ranging in age from 0–14 y. Characteristics of the children are presented in Table 1.

**Table 1:** Age and gender characteristics of the participants and proportion of children suffering from acute malnutrition.

Age		Boys	Girls	All
0–23 months	N (%)	2849 (20%)	2736 (20%)	5585 (39%)
	WHZ <-2 z-scores (%)	13%	10%	12%
24–59 months	N (%)	3147 (22%)	3031 (22%)	6178 (44%)
	WHZ <-2 z-scores (%) (%)malnutrition*	10%	9%	10%
5–7.9 years	N (%)	366 (3%)	379 (3%)	745 (5%)
	BAZ <-2 z-scores (%) (%)malnutrition**	19%	14%	16%
8–10.9 years	N (%)	451 (3%)	519 (3%)	970 (7%)
	BAZ <-2 z-scores (%) (%)malnutrition**	26%	21%	23%
11–13.9 years	N (%)	359 (3%)	320 (3%)	679 (5%)
	BAZ <-2 z-scores (%) (%)malnutrition**	40%	36%	38%
All	N (%)	7172 (51%)	6985 (51%)	14157 (100%)
	WHZ or BAZ <-2 z-scores (%) (%)malnutrition**	14%	12%	13%

Stunting increased with age with respectively 36% and 41% of children under and above 5 yrs aving HAZ <-2 z-scores. The same patterns was seen for acute malnutrition with the prevalence of WHZ<-2 z-scores and WHZ<-3 z-scores being 11% and 1% respectively in children under 5 y, whereas thinness and severe thinness were found in 26% and 5% of the children above 5 respectively. In children under 5 y, prevalence of acute malnutrition and severe acute malnutrition identified using actual WHO MUAC cut-offs of 12.5 cm and 11.5 cm were 3% and 0% respectively (Table 2).

**Table 2:** Validity of actual WHO cut-off for severe and acute malnutrition in children <5 y.

	age (y)	false positive rate	sensitivity	AUC
MUAC cut-off 12.5 cm: acute malnutrition				
boys	0–1.9 y	2.1%	17.8%	0.578
	2–4.9 y	0.2%	6.5%	0.531
girls	0–1.9 y	4.9%	32.9%	0.640
	2–4.9 y	0.3%	10.2%	0.549
all	0–1.9 y	3.5%	24.3%	0.604
	2–4.9 y	0.3%	8.1%	0.539
MUAC cut-off 11.5 cm: severe acute malnutrition				
boys	0–1.9 y	0.5%	3.3%	0.514
	2–4.9 y	0.1%	0.0%	0.500
girls	0–1.9 y	0.9%	18.2%	0.586
	2–4.9 y	0.0%	0.8%	0.540
all	0–1.9 y	0.7%	8.6%	0.540
	2–4.9 y	0.0%	2.8%	0.514

The sensitivity of MUAC ranged from 6.5% to 32.9% in children with acute malnutrition and from 0% to 18.2% in children with severe acute malnutrition with large differences between boys and girls (Table 2).

New cut-offs are presented in Table 3. When segregated into smaller age groups, sensitivity of MUAC to identify acute malnutrition increased considerably, with sensitivities from 49% to 76% for acute malnutrition, and from 55% to 83% for severe acute malnutrition. Sensitivity of the new cut-offs is higher for acute malnutrition than for severe acute malnutrition.

**Table 3:** New cut-offs by age group and gender for severe and moderate acute malnutrition for children from 0 to 14y.

age (y)	MUAC cut-off	false positive rate	specificity	sensitivity	AUC	Youden index	difference with highest Youden index	MUAC cut-off	false positive rate	specificity	sensitivity	AUC	Youden index	difference with highest Youden index	MUAC cut-off	false positive rate	specificity	sensitivity	AUC	Youden index	difference with highest Youden index*
	Boys							Girls							All						
**Acute malnutrition**																					
*All children*																					
0–1.9 y	**13.9**	32%	68%	87%	0.772	54%	0%	**13.6**	32%	68%	82%	0.746	49%	-2%	**13.7**	30%	70%	81%	0.753	51%	0%
2–4.9 y	**14.4**	30%	70%	82%	0.762	52%	-3%	**14.2**	30%	70%	84%	0.770	54%	-1%	**14.3**	30%	70%	83%	0.762	52%	-1%
5–7.9 y	**15.5**	30%	70%	84%	0.769	54%	-3%	**15.4**	33%	67%	85%	0.756	51%	-4%	**15.4**	31%	69%	84%	0.769	54%	0%
8–11.9 y	**16.4**	31%	69%	84%	0.763	53%	-1%	**16.6**	32%	68%	85%	0.764	53%	0%	**16.5**	32%	68%	83%	0.758	52%	-1%
11–13.9 y	**18.2**	38%	62%	92%	0.768	54%	0%	**17.9**	21%	79%	84%	0.813	63%	-1%	**18.2**	33%	67%	90%	0.784	57%	0%
*Stunted children*																					
0–1.9 y	**13.6**	31%	69%	85%	0.770	54%	-2%	**13.2**	31%	69%	83%	0.760	52%	-1%	**13.4**	31%	69%	80%	0.745	49%	-2%
2–4.9 y	**14**	20%	80%	78%	0.792	58%	0%	**14**	31%	69%	81%	0.750	50%	0%	**14**	25%	75%	80%	0.771	31%	0%
*Non stunted children*																					
0–1.9 y	**14**	29%	71%	85%	0.776	55%	-1%	**13.7**	32%	68%	83%	0.755	51%	0%	**13.9**	34%	66%	85%	0.756	51%	0%
2–4.9 y	N/A: no wasted non stunted children						0%							0%							0%
**Severe acute malnutrition**																					
0–1.9 y	**13.7**	29%	71%	82%	0.764	53%	-2%	**13.4**	19%	81%	73%	0.778	55%	0%	**13.5**	27%	73%	80%	0.763	53%	-1%
2–4.9 y	**14.3**	31%	69%	83%	0.760	52%	-3%	**13.8**	20%	80%	84%	0.820	64%	0%	**14.1**	27%	73%	81%	0.769	54%	-1%
5–7.9 y	**14.7**	11%	89%	58%	0.736	47%	-3%	**14.7**	17%	83%	73%	0.778	56%	-4%	**14.7**	14%	86%	63%	0.747	49%	-4%
8–11.9 y	**16**	30%	70%	92%	0.807	62%	-4%	**16.2**	31%	69%	91%	0.800	60%	-2%	**16.1**	31%	69%	91%	0.802	60%	-3%
11–13.9 y	**17.3**	25%	75%	82%	0.881	56%	-4%	**17.6**	33%	67%	89%	0.779	56%	-2%	**17.5**	31%	69%	89%	0.789	58%	0%

For new cut-offs, in children above 5y, sensitivity ranged from 58% to 94%. Youden index ranged from 31% to 64% for both acute malnutrition and severe acute malnutritionThe difference between the highest Youden index and the Youden index for the selected cut-off was between 0 and 4%.

**Fig 1:**
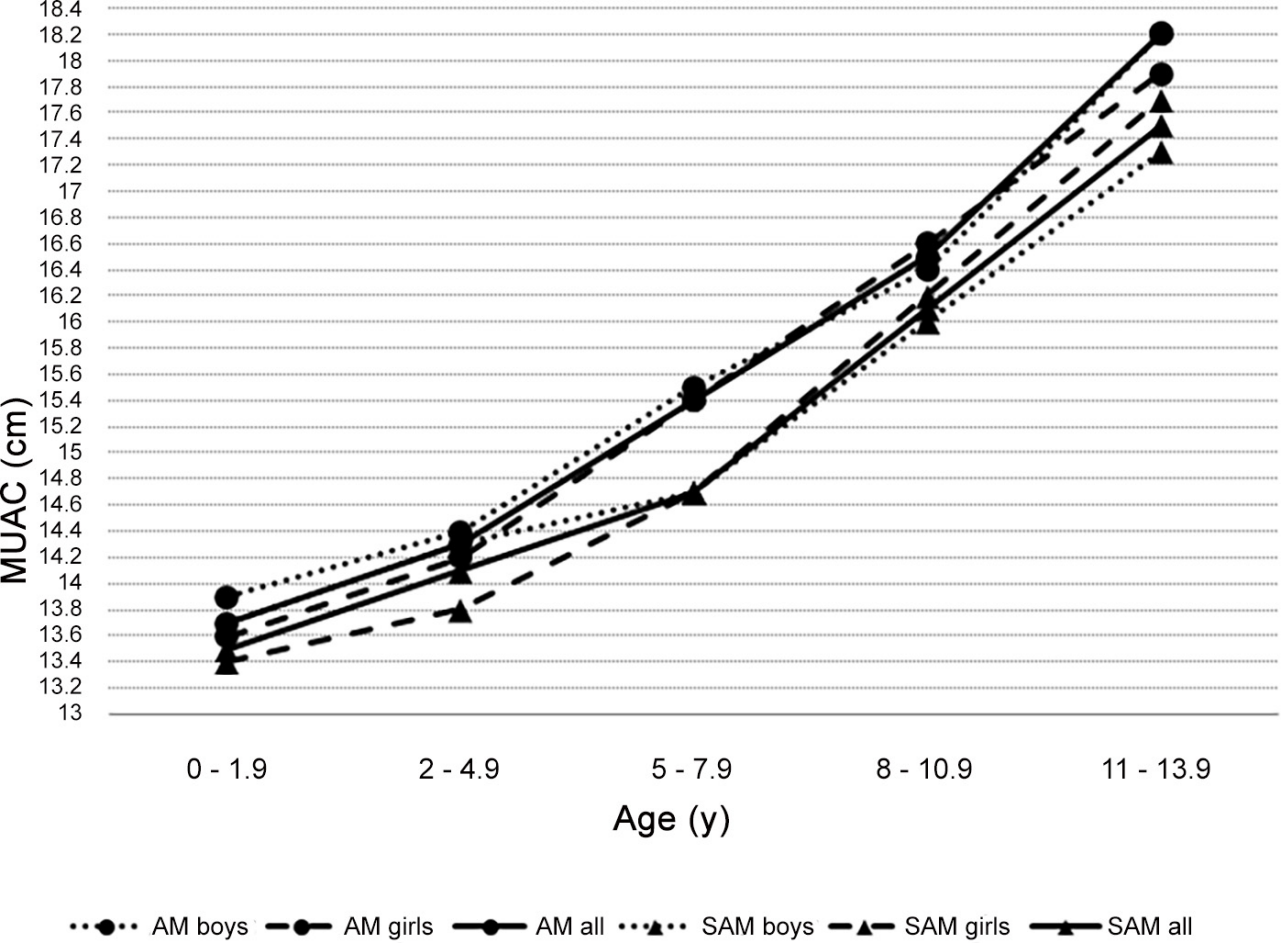
Optimal cut-offs for acute malnutrition (AM) and severe acute malnutrition (SAM) by age group and gender.

The new MUAC cut-offs for acute malnutrition and severe acute malnutrition increase with age (Fig 1) over the whole age range from 0–14 years. In both children below and above 5 y, cut-offs are generally higher for boys than for girls, but the difference between boys and girls was never more than 0.5cm and both cut-offs followed the same pattern. However, in the age group 8–10.9 y cut-off are higher in girls than in boys. The difference in new MUAC cut-off between identifying acute malnutrition (WHZ<-2) and severe acute malnutrition (WHZ<-3) for each gender and age group was small, and varied from 0.2 cm to 1 cm.

In children below 2 y, MUAC were lower in stunted children than in non-stunted children (Table 3). In the children from 2–5 years, all wasted children were also stunted, so no cut-off for stunted versus non stunted children could be calculated for this age group.

## Discussion

In this paper, optimal cut-offs for MUAC to identify acute malnutrition calculated from a pooled dataset of Cambodian children, taking age and gender into account, were higher than the current WHO recommendation. To our knowledge, this is the first paper to report optimal MUAC cut-offs in Asian children, and cut-offs for children >5 y of age. Sensitivity of identifying children with a WHZ <-2 z-score was drastically improved (from <25% to >80%) with the new cut-offs. A consequence of the higher sensitivity is of course a lower specificity, and indeed the number of false-positive cases increased also.

For example, in our dataset, 1045 wasted children (WHZ <-2 z-scores), including 155 children with WHZ <-3 z-score, were not identified as suffering from acute malnutrition by the current WHO MUAC cut-offs of 12.5cm, corresponding to 83% of wasted children below 5 y. Using the new cut-offs for gender and age as recommended in the current paper to screen for acute malnutrition, we missed only 154 wasted children (12.3%). However, using the cut-offs recommended in this paper, 4152 children would have been wrongly identified as having acute malnutrition, while using the current WHO cut-off, only 187 children would have been wrongly identified for acute malnutrition. The difference between new cut-offs for acute and severe acute malnutrition is narrow (Table 3) so we suggest to only retain cut-offs for acute malnutrition.

Half a century ago, MUAC was assumed to be almost stable between 0 and 5 years, based on well-nourished Polish children [20] but more recent studies suggested that MUAC is age and gender specific [9, 21]. A MUAC for age z-scores reference was built in 1997 but it requires knowledge of the age of the child to the nearest month, something which is often difficult to obtain in the field [14]. Therefore, the present study tried to find a balance between practicality and ideal by dividing children under 5 y in 2 groups using 2 y as a threshold.

The reliability of WHZ as a gold standard to define acute malnutrition is questioned [22]. WHZ is related to body shape and may overestimate the prevalence of acute malnutrition in some populations [23]. Also, it was reported that MUAC performs at least as well or even better than WHZ in predicting mortality among malnourished children [6, 24]. However, assessing the immediate death risk is not the only purpose of diagnosing acute malnutrition. Acute malnutrition defined by WHZ<-2 also contributes to increased morbidity, impaired physical and cognitive development, and is associated with micronutrient deficiencies [7]. Therefore acute malnutrition screening should not be reduced to mortality risk screening. Identifying only children at high risk for mortality may prevent to treat children suffering from less advanced acute malnutrition but still at high risk for impaired development. Also, we showed that in the Cambodian context, MUAC and WHZ identified a different sub-set of children as being malnourished. Hence, we have argued that both MUAC and WHZ should be used to identify malnutrition, and one cannot use one or the other [8].

However, to avoid overburdening the health system with false-positive cases of malnutrition, we suggest distinguishing 2 uses for MUAC cut-off. The first use will be the prevention of death, and the current WHO MUAC cut-off should be used to initiate treatment for severe acute malnutrition in. The newly proposed MUAC cut-offs should be used to screen for children suffering from acute malnutrition. These children may not be at immediate risk for mortality, but early detection of acute malnutrition may prevent them for later risk for mortality and for impaired development.

MUAC and WHZ are associated with different aspects of body composition, and therefore identify different groups of children with malnutrition. Therefore, both indicators should be used to start the treatment [8]. Currently, only 1 cut-off for MUAC is being recommended and used for both screening and initiation of treatment for acute malnutrition. Earlier, we have proposed to use 2 different cut-offs for MUAC: one for screening, and one for initiation of treatment for acute malnutrition [8]. The new cut-offs for acute malnutrition proposed in the present paper are meant for screening at community level for acute malnutrition, with treatment for severe acute malnutrition started using WHZ < —3 z-score as indicator of acute malnutrition or the current WHO cut-offs for MUAC. This way, sensitivity of identifying children with acute malnutrition is increased to >80%, whereas specificity for starting treatment remains the same. Using new MUAC cut-off would therefore only increase the number of children being sent to a primary health center to be re-measured for MUAC, weight and height, therefore the global cost of screening malnutrition would increase, as well as more children being correctly treated for malnutrition [21]. As obtaining a MUAC is a non-invasive procedure without any health risks, this poses not a health but an economical issue, as overtreatment can be a burden to the health system. But in return, parents of those children who are likely to be moderately acute malnourished could receive prevention counseling to avoid their children to become severe as no community management of acute malnutrition is being implemented in Cambodia at scale.

Based on the same sample of children under 5 y, Laillou et al recommend to use 13.3 cm as MUAC cut-off to screen severe acute malnutrition in children from 6 m to 5 y [8], which is lower than the recommended cut-offs in the current paper. This difference can be explained by the fact that in the current paper, the age group was split into 2 (0–1.9 y and 2–4.9 y). In addition, in the current paper, steps of 0.1 cm were used to identify the new MUAC cut-off, instead of 0.25 cm as in paper by Laillou et al. Finally, we have added the criteria of highest sensitivity and maximum acceptable false positive rate to define the new MUAC cut-off. However, although the cut-offs differ slightly, it is clear that current practice to identify children with acute malnutrition can be improved considerably by increasing the current WHO recommended cut-offs for MUAC.

Children above 5 y should also be taken into account in the management of acute malnutrition. Indeed at school age, acute malnutrition, often accompanied by micronutrient deficiencies, can delay maturation, impair muscular strength, bone density and work capacity [2]. Thus malnutrition at school age increase risk of morbidity, of school failure and school drop-out [12]. MUAC cut-offs gradually increased from birth to adolescence in an almost linear manner, but changes are influenced by changes in growth velocity, e.g. during puberty. In our study, cut-offs were higher for boys than for girls (+0.1 - +0.3 cm), except from age group 8 to 10.9 years. We assume that the earlier adolescent growth spurt in girls than in boys, with associated changes in lean and fat mass, underlies this phenomena [25]. Schools could be a practical platform to follow school-aged children for acute malnutrition.

Height of the child was another important factor in determining the new MUAC cut-offs, with lower values in stunted children compared to non stunted children (Table 3). Several studies show that stunted children tend to accumulate more fat mass and gain less lean body mass than non stunted children [26, 27], hence perhaps stunted children have lower muscle mass, leading to lower MUAC cut-offs.

A limit of the current study is that data used in this study was from Cambodia and therefore that new MUAC cut-offs presented here are only adapted to Cambodian children. Hence, the aim of the present study is not to suggest using the presented optimal cut-offs as international reference. However, the study highlights that a unique MUAC cut-off for children below 5 y results in a large of children not receiving vital treatment. Therefore we recommend a meta-analysis using the same method on a large dataset from different countries including age, gender, MUAC, height and weight to be conducted, in order to provide an international reference of MUAC cut-offs by gender and age groups to screen for acute malnutrition.

To conclude, new cut-offs for MUAC, adapted to age and gender are needed to improve the sensitivity to identify children with WHZ-score<-2 z-scores. The current WHO MUAC cut-off should be used in targeted interventions and to initiate treatment as it is adapted to detect mortality risk among malnourished children. In the present study, new MUAC cut-offs for screening for acute malnutrition, using age- and gender-specific cut-offs are presented from children from 0–14 y. Rapid adaption of these new cut-offs will result in identification of >80% of children with acute malnutrition.

## Supporting Information

S1 Fig AUC at different MUAC cut-offs level vs. acute malnutrition by gender 0–1.9 y. (PPTX)

S2 Fig AUC at different MUAC cut-offs level vs. acute malnutrition by gender 2–4.9 y. (PPTX)

S3 Fig AUC at different MUAC cut-offs level vs. acute malnutrition by gender 5–7.9 y. (PPTX)

S4 Fig AUC at different MUAC cut-offs level vs. acute malnutrition by gender 8–10.9 y. (PPTX)

S5 Fig AUC at different MUAC cut-offs level vs. acute malnutrition by gender 11–13.9 y. (PPTX)
