# Supplementary material for: Current MUAC Cut-Offs to Screen for Acute Malnutrition Need to Be Adapted to Gender and Age: The Example of Cambodia
Source: PLoS One. 2016 Feb 3;11(2):e0146442. doi: 10.1371/journal.pone.0146442 (PMC4739613; doi:10.1371/journal.pone.0146442)
Supplement: S1 Fig — (PPTX) [file pone.0146442.s001.pptx]

## Slide 1
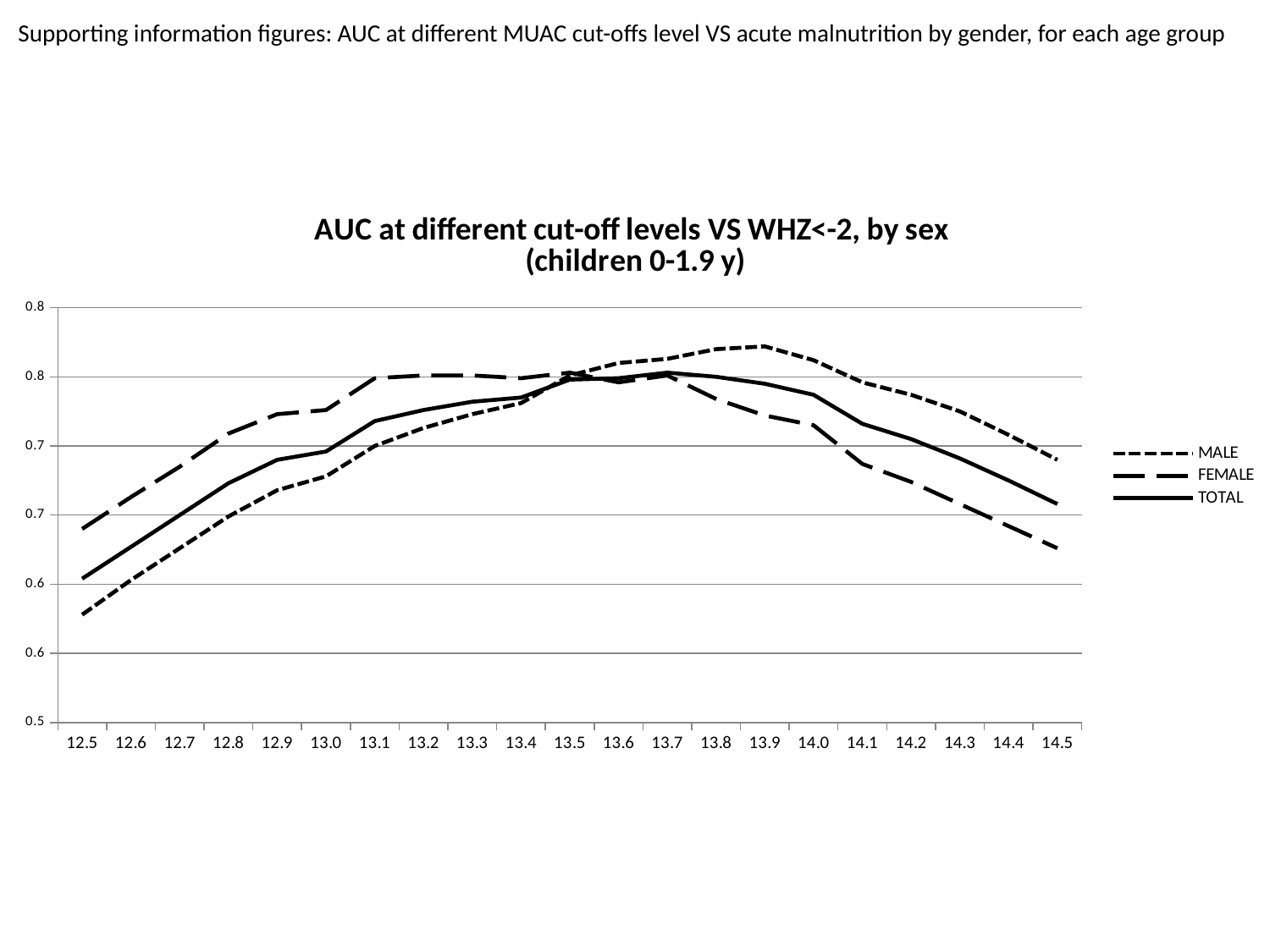

Supporting information figures: AUC at different MUAC cut-offs level VS acute malnutrition by gender, for each age group
### Chart: AUC at different cut-off levels VS WHZ<-2, by sex
(children 0-1.9 y)
| Category | MALE | FEMALE | TOTAL |
|---|---|---|---|
| 12.5 | 0.5780000000000001 | 0.6400000000000001 | 0.6040000000000001 |
| 12.6 | 0.6030000000000001 | 0.6630000000000001 | 0.6270000000000001 |
| 12.7 | 0.6260000000000001 | 0.685 | 0.6500000000000001 |
| 12.8 | 0.6490000000000001 | 0.7090000000000001 | 0.673 |
| 12.9 | 0.668 | 0.7230000000000001 | 0.6900000000000002 |
| 13 | 0.678 | 0.7260000000000001 | 0.6960000000000001 |
| 13.1 | 0.7000000000000001 | 0.7490000000000001 | 0.7180000000000001 |
| 13.2 | 0.7130000000000001 | 0.7510000000000001 | 0.7260000000000001 |
| 13.3 | 0.7230000000000001 | 0.7510000000000001 | 0.7320000000000001 |
| 13.4 | 0.7310000000000001 | 0.7490000000000001 | 0.7350000000000001 |
| 13.5 | 0.7510000000000001 | 0.7530000000000001 | 0.7480000000000001 |
| 13.6 | 0.7600000000000001 | 0.7460000000000001 | 0.7490000000000001 |
| 13.7 | 0.763 | 0.7510000000000001 | 0.7530000000000001 |
| 13.8 | 0.77 | 0.7340000000000001 | 0.7500000000000001 |
| 13.9 | 0.7719999999999999 | 0.7220000000000001 | 0.7450000000000001 |
| 14 | 0.7620000000000001 | 0.7150000000000001 | 0.7370000000000001 |
| 14.1 | 0.7460000000000001 | 0.687 | 0.7160000000000001 |
| 14.2 | 0.7370000000000001 | 0.674 | 0.7050000000000001 |
| 14.3 | 0.7250000000000001 | 0.6580000000000001 | 0.6910000000000001 |
| 14.4 | 0.7080000000000001 | 0.6420000000000001 | 0.675 |
| 14.5 | 0.6900000000000002 | 0.6260000000000001 | 0.6580000000000001 |
