# Supplementary figures and images for: Current MUAC Cut-Offs to Screen for Acute Malnutrition Need to Be Adapted to Gender and Age: The Example of Cambodia
Source: PLoS One. 2016 Feb 3;11(2):e0146442. doi: 10.1371/journal.pone.0146442 (PMC4739613; doi:10.1371/journal.pone.0146442)

## Slide 1
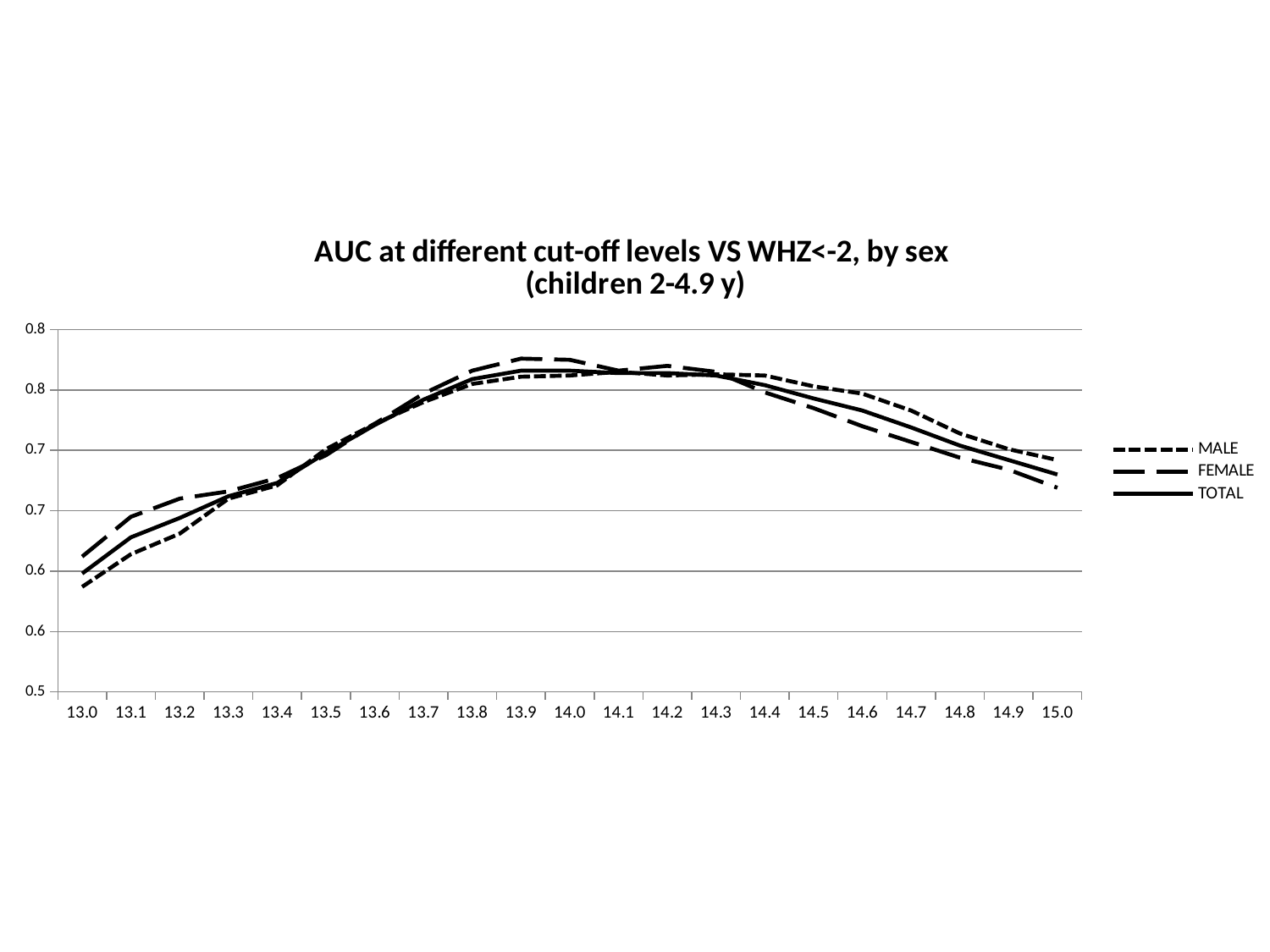

### Chart: AUC at different cut-off levels VS WHZ<-2, by sex
(children 2-4.9 y)
| Category | MALE | FEMALE | TOTAL |
|---|---|---|---|
| 13 | 0.587 | 0.6120000000000001 | 0.598 |
| 13.1 | 0.6140000000000001 | 0.6450000000000001 | 0.6280000000000001 |
| 13.2 | 0.6310000000000001 | 0.66 | 0.6440000000000001 |
| 13.3 | 0.66 | 0.666 | 0.6620000000000001 |
| 13.4 | 0.671 | 0.677 | 0.6730000000000002 |
| 13.5 | 0.7010000000000001 | 0.696 | 0.698 |
| 13.6 | 0.7220000000000001 | 0.7220000000000001 | 0.7210000000000001 |
| 13.7 | 0.7400000000000001 | 0.7470000000000001 | 0.7420000000000001 |
| 13.8 | 0.7550000000000001 | 0.7660000000000001 | 0.759 |
| 13.9 | 0.761 | 0.776 | 0.7660000000000001 |
| 14 | 0.7620000000000001 | 0.775 | 0.7660000000000001 |
| 14.1 | 0.765 | 0.7660000000000001 | 0.7640000000000001 |
| 14.2 | 0.7620000000000001 | 0.7700000000000001 | 0.7640000000000001 |
| 14.3 | 0.763 | 0.765 | 0.7620000000000001 |
| 14.4 | 0.7620000000000001 | 0.7480000000000001 | 0.7540000000000001 |
| 14.5 | 0.7530000000000001 | 0.7350000000000001 | 0.7430000000000001 |
| 14.6 | 0.7470000000000001 | 0.7200000000000001 | 0.7330000000000001 |
| 14.7 | 0.7330000000000001 | 0.7070000000000001 | 0.7190000000000001 |
| 14.8 | 0.7140000000000001 | 0.694 | 0.7040000000000001 |
| 14.9 | 0.7010000000000001 | 0.6839999999999999 | 0.692 |
| 15 | 0.692 | 0.6690000000000002 | 0.68 |

Supplement: S2 Fig — (PPTX) [file pone.0146442.s002.pptx]

## Slide 1
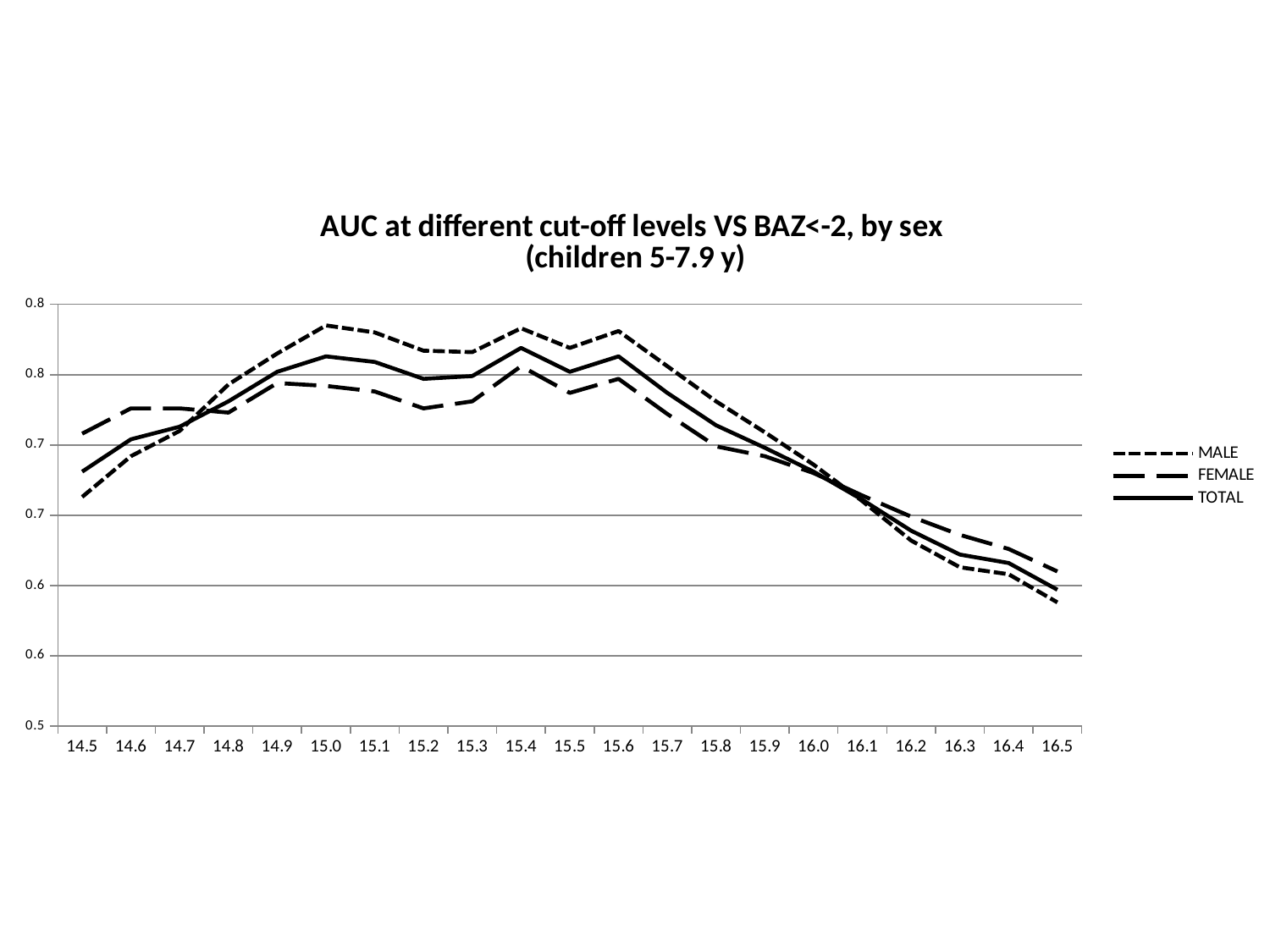

### Chart: AUC at different cut-off levels VS BAZ<-2, by sex
(children 5-7.9 y)
| Category | MALE | FEMALE | TOTAL |
|---|---|---|---|
| 14.5 | 0.6630000000000001 | 0.7080000000000001 | 0.6809999999999999 |
| 14.6 | 0.692 | 0.7260000000000001 | 0.7040000000000001 |
| 14.7 | 0.7100000000000001 | 0.7260000000000001 | 0.7130000000000001 |
| 14.8 | 0.7430000000000001 | 0.7230000000000001 | 0.7310000000000001 |
| 14.9 | 0.7650000000000001 | 0.7440000000000001 | 0.7520000000000001 |
| 15 | 0.785 | 0.7420000000000001 | 0.763 |
| 15.1 | 0.78 | 0.7380000000000001 | 0.759 |
| 15.2 | 0.7670000000000001 | 0.7260000000000001 | 0.7470000000000001 |
| 15.3 | 0.7660000000000001 | 0.7310000000000001 | 0.7490000000000001 |
| 15.4 | 0.783 | 0.7560000000000001 | 0.769 |
| 15.5 | 0.7690000000000001 | 0.7370000000000001 | 0.7520000000000001 |
| 15.6 | 0.781 | 0.7470000000000001 | 0.763 |
| 15.7 | 0.7560000000000001 | 0.7220000000000001 | 0.7370000000000001 |
| 15.8 | 0.7310000000000001 | 0.699 | 0.7140000000000001 |
| 15.9 | 0.7090000000000001 | 0.692 | 0.698 |
| 16 | 0.686 | 0.68 | 0.6809999999999999 |
| 16.100000000000001 | 0.6600000000000001 | 0.664 | 0.6610000000000001 |
| 16.2 | 0.6320000000000001 | 0.6490000000000001 | 0.6390000000000001 |
| 16.3 | 0.6130000000000001 | 0.6360000000000001 | 0.6220000000000001 |
| 16.399999999999999 | 0.6080000000000001 | 0.6260000000000001 | 0.6160000000000001 |
| 16.5 | 0.588 | 0.6100000000000001 | 0.597 |

Supplement: S3 Fig — (PPTX) [file pone.0146442.s003.pptx]

## Slide 1
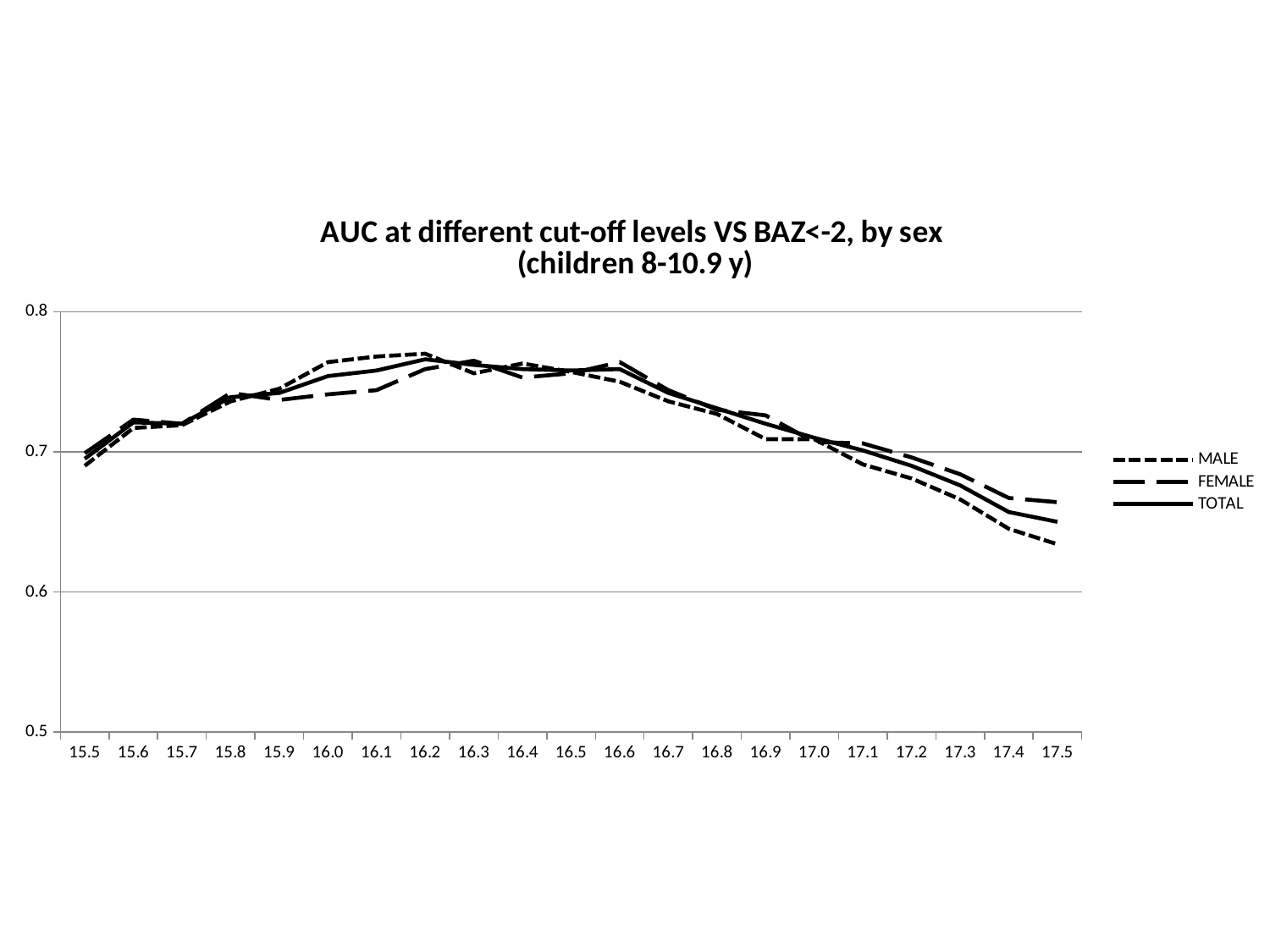

### Chart: AUC at different cut-off levels VS BAZ<-2, by sex
(children 8-10.9 y)
| Category | MALE | FEMALE | TOTAL |
|---|---|---|---|
| 15.5 | 0.6900000000000001 | 0.699 | 0.695 |
| 15.6 | 0.7170000000000001 | 0.7230000000000001 | 0.7210000000000001 |
| 15.7 | 0.7190000000000001 | 0.7200000000000001 | 0.7200000000000001 |
| 15.8 | 0.7360000000000001 | 0.7420000000000001 | 0.7390000000000001 |
| 15.9 | 0.7450000000000001 | 0.7370000000000001 | 0.7420000000000001 |
| 16 | 0.7640000000000001 | 0.7410000000000001 | 0.7540000000000001 |
| 16.100000000000001 | 0.7680000000000001 | 0.7440000000000001 | 0.7580000000000001 |
| 16.2 | 0.7700000000000001 | 0.759 | 0.7660000000000001 |
| 16.3 | 0.7560000000000001 | 0.765 | 0.7620000000000001 |
| 16.399999999999999 | 0.763 | 0.7530000000000001 | 0.759 |
| 16.5 | 0.7570000000000001 | 0.7560000000000001 | 0.7580000000000001 |
| 16.600000000000001 | 0.7500000000000001 | 0.7640000000000001 | 0.759 |
| 16.7 | 0.7360000000000001 | 0.7440000000000001 | 0.7420000000000001 |
| 16.8 | 0.7270000000000001 | 0.7300000000000001 | 0.7310000000000001 |
| 16.899999999999999 | 0.7090000000000001 | 0.7260000000000001 | 0.7200000000000001 |
| 17 | 0.7090000000000001 | 0.7070000000000001 | 0.7100000000000001 |
| 17.100000000000001 | 0.691 | 0.7060000000000001 | 0.7010000000000001 |
| 17.2 | 0.6809999999999999 | 0.696 | 0.6900000000000001 |
| 17.3 | 0.666 | 0.684 | 0.676 |
| 17.399999999999999 | 0.6450000000000001 | 0.6670000000000001 | 0.6570000000000001 |
| 17.5 | 0.6340000000000001 | 0.6640000000000001 | 0.6500000000000001 |

Supplement: S4 Fig — (PPTX) [file pone.0146442.s004.pptx]

## Slide 1
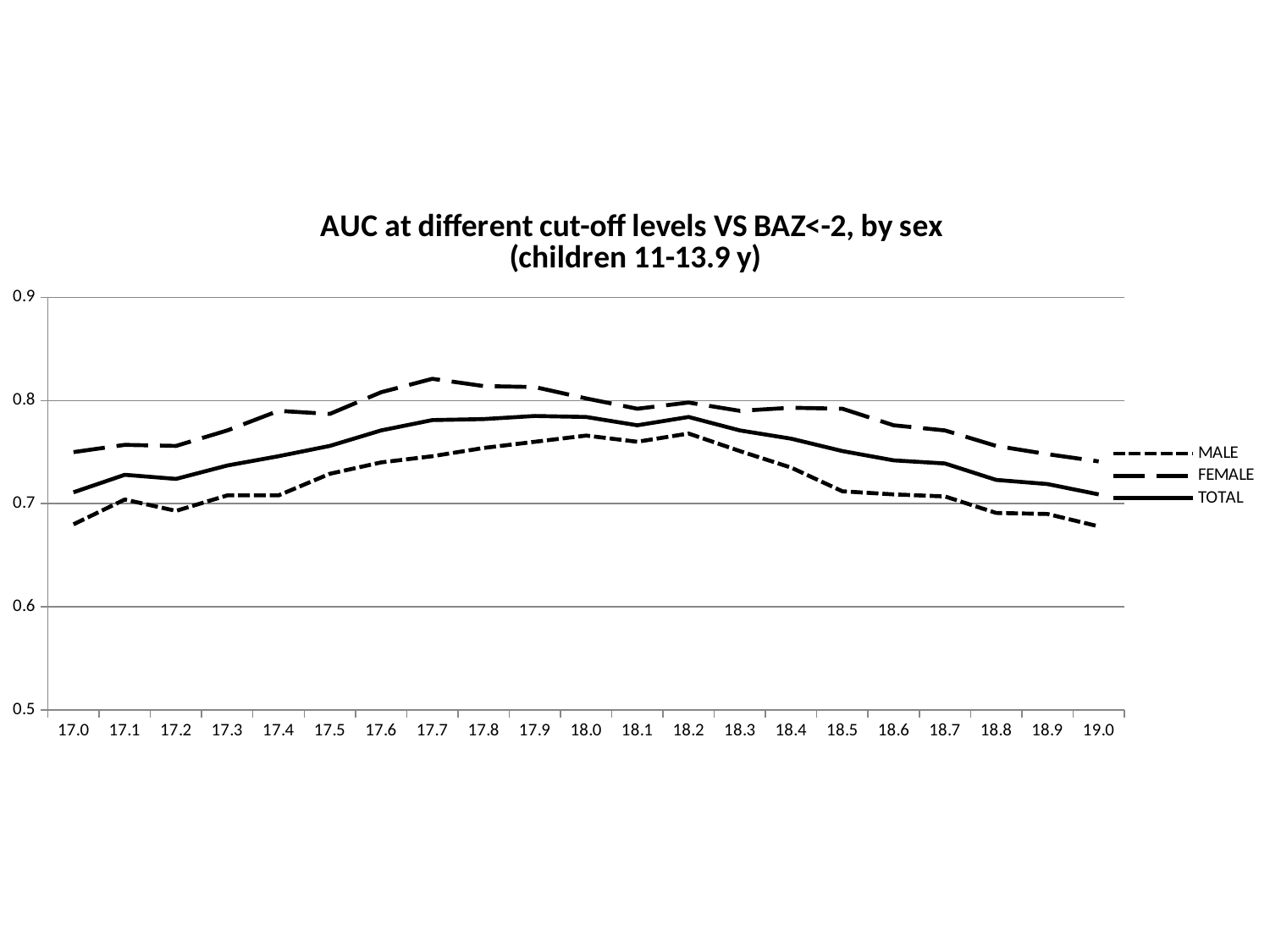

### Chart: AUC at different cut-off levels VS BAZ<-2, by sex
(children 11-13.9 y)
| Category | MALE | FEMALE | TOTAL |
|---|---|---|---|
| 17 | 0.68 | 0.7500000000000001 | 0.7110000000000001 |
| 17.100000000000001 | 0.7040000000000001 | 0.7570000000000001 | 0.7280000000000001 |
| 17.2 | 0.693 | 0.7560000000000001 | 0.7240000000000001 |
| 17.3 | 0.7080000000000001 | 0.771 | 0.7370000000000001 |
| 17.399999999999999 | 0.7080000000000001 | 0.7899999999999999 | 0.7460000000000001 |
| 17.5 | 0.7290000000000001 | 0.7869999999999999 | 0.7560000000000001 |
| 17.600000000000001 | 0.7400000000000001 | 0.808 | 0.771 |
| 17.7 | 0.7460000000000001 | 0.8210000000000002 | 0.7809999999999999 |
| 17.8 | 0.7540000000000001 | 0.8140000000000002 | 0.7819999999999999 |
| 17.899999999999999 | 0.7600000000000001 | 0.8130000000000002 | 0.7849999999999999 |
| 18 | 0.7660000000000001 | 0.802 | 0.7839999999999999 |
| 18.100000000000001 | 0.7600000000000001 | 0.7919999999999999 | 0.776 |
| 18.2 | 0.7680000000000001 | 0.7979999999999999 | 0.7839999999999999 |
| 18.3 | 0.7510000000000001 | 0.7899999999999999 | 0.771 |
| 18.399999999999999 | 0.7350000000000001 | 0.7929999999999999 | 0.763 |
| 18.5 | 0.7120000000000001 | 0.7919999999999999 | 0.7510000000000001 |
| 18.600000000000001 | 0.7090000000000001 | 0.776 | 0.7420000000000001 |
| 18.7 | 0.7070000000000001 | 0.771 | 0.7390000000000001 |
| 18.8 | 0.691 | 0.7560000000000001 | 0.7230000000000001 |
| 18.899999999999999 | 0.6900000000000001 | 0.7480000000000001 | 0.7190000000000001 |
| 19 | 0.678 | 0.7410000000000001 | 0.7090000000000001 |

Supplement: S5 Fig — (PPTX) [file pone.0146442.s005.pptx]
